# Linguistic difference in the effect of organized programs on socioeconomic inequalities in breast cancer screening: ecological study in Switzerland

**DOI:** 10.1097/CEJ.0000000000000914

**Published:** 2024-08-05

**Authors:** Clement P. Buclin, Martina von Arx, Vladimir Jolidon, José Luis Sandoval, Fabienne Buholzer-Mercier, Justine E. Daverio, Bernadette W.A. van der Linden, Philippe Wanner, Idris Guessous, Delphine S. Courvoisier, Stéphane Cullati

**Affiliations:** a Department of Internal Medicine, Geneva University Hospitals; b Institute of Sociological Research, University of Geneva, Geneva, Switzerland; c School of International Business and Marketing, University of Economics, Ho Chi Minh City, Vietnam; d Division of Primary Care, Department of Health and Community Medicine, Geneva University Hospitals and Faculty of Medicine, University of Geneva; e Division of Oncology, Department of Oncology, Geneva University Hospitals, Geneva; f Department of Community Health, Population Health Laboratory (#PopHealthLab), University of Fribourg, Fribourg; g Quality of care division, Medical directorate, Geneva University Hospitals; h Institute of Demography and Socioeconomics, University of Geneva, Geneva, Switzerland

**Keywords:** breast cancer screening, opportunistic screening, organized screening, prevention, socioeconomic inequalities

## Abstract

**Objective:**

The objective of this study is to examine how the effect of organized mammography screening programs on breast cancer screening participation differ between socioeconomic strata and how this relationship may be modified by the context of linguistic differences. Switzerland, marked by its diverse linguistic landscape, reflects cultural variations alongside differences in public health strategies. The goal of this study was to assess potential socioeconomic differences in regional mammography screening programs effectiveness to improve breast cancer screening participation.

**Methods:**

Data on 14 173 women in the regionally adapted breast cancer screening age range was drawn from five cross-sectional waves of the nationally representative Swiss Health Interview Survey (1997–2017). Socioeconomic indicators included education, household income, and employment status. Poisson regression was used to estimate the adjusted prevalence ratios of up-to-date (last 2 years) mammography uptake. Inequality was assessed using relative index of inequality and the slope index of inequality.

**Results:**

Organized screening programs were generally effective and increased up-to-date mammography uptake by close to 20 percentage points in both regions. While in the Latin cantons, screening programs had no impact on socioeconomic inequalities in screening, it reduced inequalities for women with lower education in the German cantons. This modification effect of screening programs was not seen for income and employment-related inequalities and did not differ across linguistic regions.

**Conclusions:**

Public health agencies should consider the different cultural reception of programs as addressing these differences could help ensure that breast cancer screening initiatives are not only effective, but also culturally equitable across different socioeconomic groups.

## Introduction

From 2002 onwards, overall breast cancer mortality declined in Europe (Carioli *et al.*, 2017), which is partly due to the implementation of organized screening programs for women over 50 (Marmot *et al.*, 2013). The majority of Switzerland’s neighboring countries have ongoing population-based breast cancer screening programs (Deandrea *et al.*, 2016; Feller *et al.*, 2017) and general breast cancer screening rates of about 50% (Malek and Kääb-Sanyal, 2016; Quintin *et al.*, 2022). Although Switzerland shows similar rates of mammography to its neighboring countries, there are variable breast cancer screening activities across its regions (Fenner *et al.*, 2018).

Contrary to France and Germany, which have nationwide breast-screening programs, Switzerland delegates the responsibility for implementing mammography screening programs to its 26 administrative regions (cantons) (Supplementary Figure 1, Supplemental Digital Content 1, http://links.lww.com/EJCP/A489). Starting in 1999, almost all French- and Italian-speaking regions (hereafter, ‘Latin regions’) have implemented regional screening programs, meaning population-based programs inviting people reaching the target age, since 1999. Across the German-speaking cantons (hereafter ‘German regions’), opportunistic screening, that is, screening proposed by a doctor initiated by patients themselves, still dominates (Cullati *et al.*, 2018). A cross-sectional analysis of mammography attendance from 2012 revealed that screening rates in the 2 years before (hereafter, ‘up-to-date mammography screening’) were twice as high in regions with organized screening compared to regions with opportunistic screening (Eichholzer *et al.*, 2016).

Research has evidenced higher breast cancer incidence among wealthy women compared to those from lower socioeconomic backgrounds (Lundqvist *et al.*, 2016). However, studies have shown that this discrepancy is primarily due to underdiagnosis among the socioeconomically disadvantaged (Damiani *et al.*, 2015; Mottram *et al.*, 2021). This can be explained by the fact that individuals with advantages socioeconomic position tend to undergo more screening compared to disadvantaged individuals. Population-based cancer screening programs aim to promote egalitarian access to early detection and ultimately reduce socioeconomic inequalities in screening uptake. Nevertheless, research has shown inconsistent populational effects of breast cancer screening programs across socioeconomic strata. While these programs increase coverage within hard-to-reach populations or women with low educational achievement (Espinas *et al.*, 2011), they also increase it among women from high socioeconomic groups, thus exacerbating existing socioeconomic inequalities (Aarts *et al.*, 2011). In addition, research has shown that organized programs had inconsistent effects on socioeconomic disparities in breast cancer screening rates in multilingual countries, like Belgium (Renard *et al.*, 2014; Willems and Bracke, 2018) and Switzerland (Sandoval *et al.*, 2017; Jolidon *et al.*, 2022).

Switzerland has two distinct cultural region that are primarily differentiated by their linguistic differences. The German speaking region and the Latin region (French and Italian speaking) show consistent differences in most sociocultural indicators, notably in their attitudes toward family, community, and cultural behaviors (Laesser *et al.*, 2014; Cheval *et al.*, 2019). These cultural differences also affect health behaviors in Switzerland’s linguistic regions, impacting smoking (Richard *et al.*, 2016), dietary patterns (Richard *et al.*, 2016; Chatelan *et al.*, 2017), and vaccination uptake (Altpeter *et al.*, 2018; Seiler *et al.*, 2021). The linguistic region of residence is associated with differences in cancer-protective lifestyle (Suter *et al.*, 2023), intention to undergo mammography (Labrie *et al.*, 2017) and with rates of prostate (Bähler *et al.*, 2021) and cervical (Burton-Jeangros *et al.*, 2017) cancer screening. Therefore, the linguistic region of residence may modify the effects of mammography screening programs on socioeconomic inequalities in up-to-date mammography screening.

Hence, this study aims to determine if the linguistic region of residence modified the impact of organized screening programs on socioeconomic inequalities in up-to-date mammography.

## Methods

Data from the cross-sectional population-based Swiss Health Interview Survey (SHIS) spanning 1997–2017 were analyzed. The SHIS is nationwide survey focusing on health status and health-related behavior in Switzerland. The survey aimed to draw a representative sample of the Swiss population in terms of age, gender, socioeconomic status, and geographic region. The stratification by geographic region ensured that all regions of Switzerland were proportionally represented in the sample. The inclusion criteria were designed to reflect the demographic composition of the Swiss population, focusing on individuals living in private households and fluent in one of the three national languages. The design of this survey has been further described elsewhere (Volken, 2013). Participants with missing data were excluded. The study population was restricted to women within the regionally adapted breast cancer screening age range, corresponding to 50–69 years (Cornuz *et al.*, 2015; Schünemann *et al.*, 2020) – except three regions where the upper limit ranged until 70 or 74 (See Supplementary Table 1, Supplemental Digital Content 1, http://links.lww.com/EJCP/A489). Among survey participants, 10 792 (84.5%) out of 12 769 accepted the interview in 1997; 16 141 (86%) out of 18 759 in 2002; 18 760 (66%) out of 28 319 in 2007; 21 597 (53%) out of 41 008 in 2012; and 22 134 (51%) out of 43 769 in 2017. This subsumed in an overall response rate of 68%. Of 89 454 respondents, 14 173 were eligible women.

### Dependent variable

Participants were asked if they had ever undergone a mammogram and, if affirmative, the date of their most recent mammogram allowing to compute the variable of up-to-date mammography screening.

### Independent variables

Socioeconomic inequalities were measured through three socioeconomic class (SEC) indicators: (1) educational level according to the International Standard Classification of Education (primary, secondary, tertiary); (2) monthly household income in CHF (1 CHF = 1.09 USD in 2012) weighted with the number of persons living in the household and the number of children under 14 years old (Mack *et al.*, 2020) (≤2000, 2001–4000, 4001–6000, >6000); (3) employment (full time, part-time, and without employment).

### Modifier variables

Modifiers were mammography screening programs and linguistic regions. Women’s exposition to mammography screening programs was determined based on their residency in different Swiss cantons. A full list of Swiss cantons’ organized mammography programs as well as the age range considered is available in supplementary materials (Supplementary Table 1, Supplemental Digital Content 1, http://links.lww.com/EJCP/A489) and more details on their implementations are published elsewhere (Bulliard *et al.*, 2021).

We identified the language of the respondents using the language of the respondent’s municipality of residence. The three languages, German, French, and Italian, were categorized into German or Latin (French and Italian). This categorization was based on the cultural similarities between French and Italian cultures and their difference from German culture. Previous work showed that Latin countries tend to have a more relaxed relationship with authority compared to Germany, where rule-following is more prominent (Hofstede, 2001; Chhokar *et al.*, 2007). Language was considered as a proxy of culture (Altarriba and Basnight-Brown, 2022).

### Control variables

Due to their potential association with mammography uptake (Maruthur *et al.*, 2009; Martin-Lopez *et al.*, 2013; Carrasco-Garrido *et al.*, 2014; Kang *et al.*, 2014), control variables were: age, marital status, current smoker, self-rated health, number of general practitioner, and gynecologist visits in the last 12 months.

### Statistical analysis

The prevalence of up-to-date mammography screening was corrected for sampling and nonparticipation bias using a weight variable available in the SHIS dataset.

We examined the association between SEC and up-to-date mammography screening at the individual level with adjusted prevalence ratio (APR) estimated with Poisson regression and reported with 95% confidence intervals (CI). The directed acyclic graph guiding our hypothesis is depicted in Supplementary Figure 2, Supplemental Digital Content 1, http://links.lww.com/EJCP/A489.

Robust variance estimators were used to relax the assumption that the outcome distribution follows a Poisson distribution. Collinearity between socioeconomic conditions’ variables was tested with variance inflation factor, and no evidence of collinearity was found. Results of multicollinearity test are available upon request to the authors. This model was stratified by organized mammography screening programs and linguistic regions, resulting in four different models. In addition, the same model was used in each linguistic region with the addition of an interaction term between organized mammography screening programs and each SEC variable, resulting in three models per region.

We conducted an additional sensitivity analysis using the relative index of inequality (RII) and the slope index of inequality (SII) for education and household income. The RII and SII estimate the linear association across the entire socioeconomic scale considering the relative size of each SEC subgroup (Moreno-Betancur *et al.*, 2015). All analyses were conducted with STATA 17 including the RIIGEN package.

## Results

The mean age of responding women was 59.3 years in both the German and the Latin regions (Table 1 and Supplementary Table 2, Supplemental Digital Content 1, http://links.lww.com/EJCP/A489).

**Table 1:** Characteristics of 14 173 women aged 50–69 years and proportions of up-to-date mammography screening, by linguistic region (German versus Latin), Switzerland (SHIS), 1997–2017

	Characteristics of women			Characteristics of women with up-to-date mammography screening		
	German regions	Latin regions		German regions	Latin regions	
	*n* = 9076	*n* = 5097		*n* = 3290	*n* = 3420	
	*n* (%^a^)	*n* (%^a^)	*P*-value^b^	*n* (%^a^)	*n* (%^a^)	*P*-value^b^
Education			<0.001			<0.001
Primary	1791 (19)	1355 (26)		590 (17)	869 (25)	
Secondary	5855 (65)	2840 (55)		2187 (67)	1901 (55)	
Tertiary	1430 (16)	902 (19)		513 (16)	650 (20)	
Monthly household income			<0.001			<0.001
1^st^ quintile	1382 (15)	1094 (21)		450 (14)	653 (19)	
2^nd^ quintile	1652 (18)	1073 (21)		557 (17)	721 (21)	
3^rd^ quintile	2159 (24)	1102 (22)		795 (25)	758 (22)	
4^th^ quintile	2203 (24)	1010 (19)		817 (24)	702 (20)	
5^th^ quintile	1680 (18)	818 (16)		671 (2)	586 (17)	
Employment			<0.001			<0.001
Full-time employment	1562 (16)	1037 (20)		578 (17)	698 (20)	
Part-time employment	3686 (42)	1476 (31)		1363 (43)	1040 (32)	
Unemployed	101 (1)	91 (2)		32 (1)	50 (1)	
Out of the labor force	3727 (41)	2493 (48)		1317 (39)	1632 (46)	

Household income is weighted according to the OECD-modified equivalence scale.

SHIS, Swiss Health Interview Survey.

a Proportions are weighted for sampling strategy and response bias.

b Chi-square test, unweighted.

Between 1997 and 2017, the prevalence of up-to-date mammography was 36.3% for women living in the German region and 67.1% for women living in the Latin region. The regional differences in the distribution of socioeconomic characteristics of women with up-to-date screening were similar to those of the overall sample (Table 1). The prevalence of up-to-date mammography screening by socioeconomic characteristics stratified by mammography screening programs and linguistic regions is reported in Supplementary Table 3, Supplemental Digital Content 1, http://links.lww.com/EJCP/A489.

### German region

The weighted prevalence of up-to-date mammography among women living in the German region with opportunistic screening was 33.5% and with organized screening programs was 50.8% (Table 2).

**Table 2:** Socioeconomic conditions and adjusted prevalence ratios of up-to-date mammography screening, by linguistic region and mammography program implementation, weighted, women aged 50–69, Switzerland, 1997–2017

	Women living in cantons with		
	No mammography program (opportunistic screening)	Regional mammography program implemented	*P*-value for the interaction between mammography program exposure and socioeconomic status variables^b^
	**German region** ^c^		
	*n* = 7582	*n* = 1494	*n* = 9076
Prevalence (weighted)^a^	33.5%	50.8%	
	APR [95% CI]	APR [95% CI]	*P*-value
Education (ref.: primary)			0.017
Secondary	1.13* (1.01–1.26)	0.95 (0.81–1.12)	
Tertiary	1.07 (0.92–1.24)	0.80* (0.64–0.99)	
Household income (ref.: 1^st^ quintile)			0.429
2^nd^ quintile	1.11 (0.95–1.29)	1.01 (0.83–1.22)	
3^rd^ quintile	1.14 (1.00–1.31)	1.19 (0.99–1.44)	
4^th^ quintile	1.17* (1.02–1.35)	1.09 (0.89–1.30)	
5^th^ quintile	1.20* (1.03–1.39)	1.20 (0.96–1.48)	
Employment (ref: full-time)			0.506
Part-time employment	1.01 (0.90–1.13)	0.93 (0.78–1.10)	
Out of the labour force	0.98 (0.85–1.11)	0.94 (0.76–1.15)	
Unemployed	1.06 (0.74–1.51)	0.99 (0.50–1.96)	
	**Latin region** ^d^		
	*n* = 1881	*n* = 3216	*n* = 5097
Prevalence (weighted)^a^	54.6%	74.6%	
	APR [95% CI]	APR [95% CI]	*P*-value
Education (ref. primary)			0.451
Secondary	0.97 (0.86–1.10)	0.99 (0.94–1.06)	
Tertiary	0.95 (0.78–1.15)	1.02 (0.95–1.09)	
Household income (ref.: 1^st^ quintile)			0.731
2^nd^ quintile	1.03 (0.88–1.21)	1.15*** (1.06–1.25)	
3^rd^ quintile	1.01 (0.86–1.19)	1.16** (1.07–1.26)	
4^th^ quintile	1.06 (0.88–1.26)	1.15** (1.06–1.25)	
5^th^ quintile	1.11 (0.92–1.34)	1.19*** (1.09–1.29)	
Employment (ref: full-time)			0.489
Part-time employment	0.93 (0.80–1.09)	1.02 (0.95–1.09)	
Out of the labour force	1.00 (0.86–1.17)	0.97 (0.90–1.04)	
Unemployed	0.89 (0.58–1.36)	0.89 (0.71–1.11)	

APRs are weighted for sampling frame and response bias. Household income is weighted according to the OECD-modified equivalence scale. Variables used for adjustment: age, marital status, currently smoking, self-rated health, number of general practitioner and gynecologist visits in the last 12 months, and time (survey wave dummies).

APR, adjusted prevalence ratios.

a Survey weights were used to correct for sampling frame and response bias.

b Obtained by adding an interaction term between the socioeconomic status and mammography program variable (in separate models for each socioeconomic status variable).

c Aargau, Appenzell Ausserrhoden, Appenzelle Innerrhoden, Basel-Stadt, Basel-Landschaft, Bern, Glarus, Graubünden, Luzern, Nidwalden, Obwalden, Schaffhausen, Schwyz, Solothurn, St-Gallen, Thurgau, Uri, Zug, and Zurich.

d Geneva, Fribourg, Jura, Neuchâtel, Ticino, Valais, and Vaud.

* *P* ≤ 0.05

** *P* ≤ 0.01

*** *P* ≤ 0.001.

In cantons with opportunistic screening, the prevalence of up-to-date mammography was higher among women with secondary education (APR 1.13, 95% CI [1.01–1.26]). This was also the case for women in the fourth (APR 1.17, 95% CI [1.02–1.35]) and fifth (APR 1.20, 95% CI [1.03–1.39]) quintiles of household income compared to women in the first income quintile (Table 2). Using the RII and the SII indices (Table 3), the prevalence of up-to-date mammography did not differ by education (RII APR = 1.10, 95% CI [0.92–1.31]; SII *b* = 0.03, 95% CI [−0.03 to 0.09]) but it did differ by household income (RII APR = 1.21, 95% CI [1.04–1.41]; SII *b* = 0.06, 95% CI [0.01–0.11]).

**Table 3:** Relative index of inequality and slope index of inequality for education and household income in up-to-date mammography screening by linguistic region and mammography program implementation, weighted, women aged 50–69, Switzerland 1997–2017

	Women living in cantons with	
	No mammography program (opportunistic screening)	Regional mammography program implemented
	**German region** ^a^	
	*n* = 7582	*n* = 1494
RII	APR [95% CI]	APR [95% CI]
Education	1.10 (0.92 to 1.31)	0.77* (0.59 to 0.99)
Household income	1.21* (1.04 to 1.41)	1.21 (0.96 to 1.53)
SII	*b* [95% CI]	*b* [95% CI]
Education	0.03 (−0.03 to 0.09)	−0.13* (−0.26 to 0.00)
Household income	0.06* (0.01 to 0.11)	0.10 (−0.02 to 0.21)
	**Latin region** ^b^	
	*n* = 1881	*n* = 3216
RII	APR [95% CI]	APR [95% CI]
Education	0.93 (0.75 to 1.16)	1.02 (0.93 to 1.12)
Household income	1.11 (0.91 to 1.37)	1.17*** (1.07 to 1.28)
SII	*b* [95% CI]	*b* [95% CI]
Education	−0.04 (−0.16 to 0.09)	0.01 (−0.05 to 0.08)
Household income	0.06 (−0.06 to 0.16)	0.13*** (0.05 to 0.18)

RII and SII are weighted for sampling frame and response bias. Household income is weighted according to the OECD-modified equivalence scale. Variables used for adjustment: age, marital status, currently smoking, self-rated health, number of general practitioner and gynecologist visits in the last 12 months, and time (survey wave dummies).

RII, relative index of inequality; SII, slope index of inequality.

a Aargau, Appenzell Ausserrhoden, Appenzelle Innerrhoden, Basel-Stadt, Basel-Landschaft, Bern, Glarus, Graubünden, Luzern, Nidwalden, Obwalden, Schaffhausen, Schwyz, Solothurn, St-Gallen, Thurgau, Uri, Zug, and Zurich.

b Geneva, Fribourg, Jura, Neuchâtel, Ticino, Valais, and Vaud.

* *P* ≤ 0.05.

*** *P* ≤ 0.001.

In cantons with mammography screening programs, the prevalence of up-to-date mammography was lower among women with tertiary education compared to women with primary education (APR 0.80, 95% CI [0.64–0.99]). Using the RII and SII indices (Table 3), the prevalence of up-to-date mammography differed by education (RII APR = 0.76, 95% CI [0.59–0.99]; SII *b* = −0.13, 95% CI [−0.26 to 0.00]) but not by household income (RII APR = 1.21, 95% CI [0.96–1.53]; SII *b* = 0.10, 95% CI [−0.02 to  0.21]).

The interaction between education and exposure to a mammography screening program was significant. Hence, mammography screening programs modified the association between education and the prevalence of up-to-date mammography screening. However, incorporating the interaction term increased the Quasi Information Criterion of the model from 4104 to 4133, indicating no improvement in model fit compared to the model without the interaction term.

**Fig. 1:**
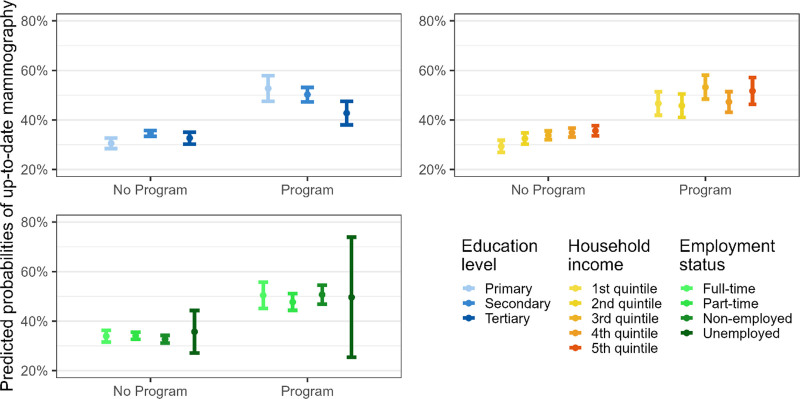
Predicted probabilities of up-to-date mammography screening by socioeconomic variables in regions with/without a mammography program, German region, Switzerland, 1997–2017. Predicted probabilities are derived from the models with interaction terms in Table 2. Confidence intervals are computed using a multiplier of 1.39 standard errors because two parameters are compared and not a parameter with a fixed point (Goldstein H, Healy MJR. The graphical presentation of a collection of means. J R Statist Soc. (1995) 158:175–7).

In cantons with mammography programs, the predicted probability of up-to-date mammography was higher among women with primary education than among women with tertiary education, suggesting that the impact of the mammography programs was more important among women with primary education than in women with tertiary education (Fig. 1 and Fig. 3).

### Latin region

Among women living in the Latin region, the weighted prevalence of up-to-date mammography was 54.6% for those living in cantons with opportunistic screening and 74.6% in cantons with organized screening programs. In cantons with opportunistic screening, there were no differences in the prevalence of up-to-date mammography by educational level, household income, and employment status (Table 2). Using the RII and SII indices (Table 3), the prevalence of mammography screening did not differ by educational level (RII: APR = 1.11, 95% CI [0.75–1.16]; SII *b* = −0.04, 95% CI [−0.16 to 0.09]) or household income for the RII (APR = 1.11, 95% CI [0.91–1.37]) and the SII (*b* = 0.06, 95% CI [−0.06 to 0.16]).

In cantons with mammography screening programs, the prevalence of up-to-date mammography was higher among women in the second (APR 1.15, 95% CI [1.06–1.25]), third (APR 1.16, 95% CI [1.07–1.26]), fourth (APR 1.15, 95% CI [1.06–1.25]), and fifth (APR 1.19, 95% CI [1.09–1.29]) quintiles of household income (Table 2). No differences were observed by educational level or employment status. Using the RII and SII indices (Table 3), the prevalence of mammography screening did not differ by educational level (RII APR = 1.02, 95% CI [0.93–1.12]; SII *b* = 0.01, 95% CI [−0.05 to 0.08]), but did differ by household income (RII APR = 1.17, 95% CI [1.07–1.28]; SII *b* = 0.13, 95% CI [0.05–0.18]).

**Fig. 2:**
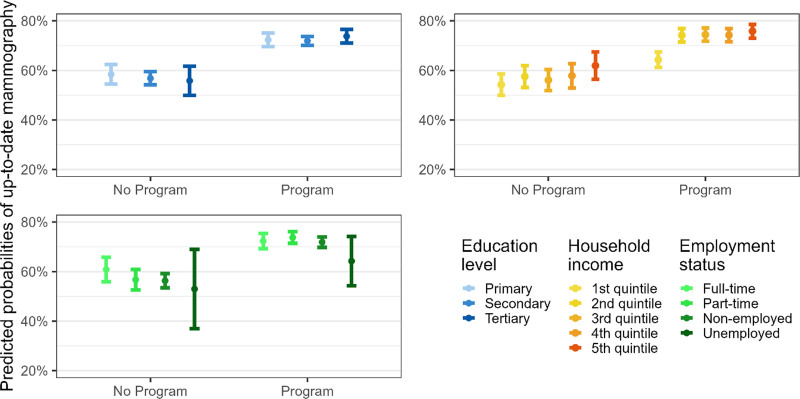
Predicted probabilities of up-to-date mammography screening by socioeconomic variables in regions with/without a mammography program, Latin region, Switzerland, 1997–2017. Predicted probabilities are derived from the models with interaction terms in Table 2. Confidence intervals are computed using a multiplier of 1.39 standard errors because two parameters are compared and not a parameter with a fixed point (Goldstein H, Healy MJR. The graphical presentation of a collection of means. J R Statist Soc. (1995) 158:175–7).

**Fig. 3:**
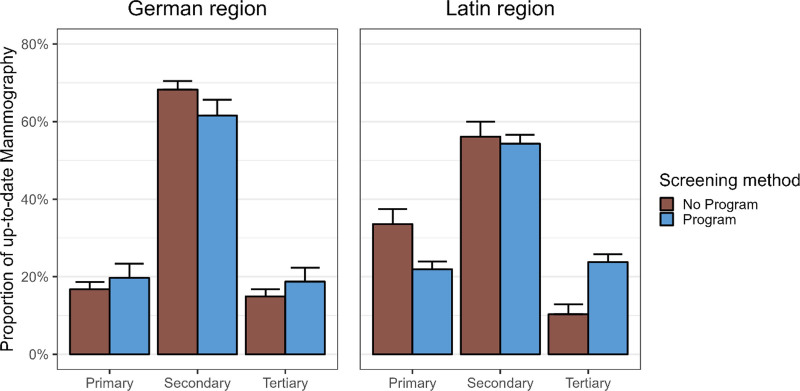
Observed proportions with 95% confidence intervals of up-to-date mammography screening by educational level and by exposure to with/without a mammography program in the two linguistic regions (German versus Latin), Switzerland, 1997–2017.

The interactions between socioeconomic conditions and exposure to a mammography screening program were not significant, meaning that mammography screening programs did not modify the association between socioeconomic conditions and the prevalence of up-to-date mammography screening (Fig. 2 and Fig. 3).

## Discussion

This study investigated whether the impact of mammography screening programs on socioeconomic inequalities in up-to-date mammography screening differed between the linguistic areas of Switzerland, namely the Latin and German regions. Results showed that the overall prevalence of up-to-date mammography was higher in the Latin than in the German region and lower in cantons relying on opportunistic screening than in cantons with screening programs (around 20% absolute difference). These results are consistent with similar findings from a study in Belgium which also showed regional differences regarding the increase of mammography rates over time depending on their screening strategy (Willems and Bracke, 2018). Similarly to Switzerland, Belgium is a multicultural country with regions that speak Germanic languages (Flemish and German) and a region that speaks a Latin language (French). The Belgian study showed that implementation of the mammography program in the French speaking region suffered a setback after 2008, which they attributed to growing controversy surrounding mammograms. However, this controversy does not fully explain why this setback was only observed in the Latin region. Our study proposes a further explanation for this phenomenon by showing that German and Latin culture, as evaluated by the language spoken, might react differently to organized screening programs in multicultural countries.

In the German region with opportunistic screening, we observed socioeconomic inequalities regarding household income and education level, with higher attendance rates among women with higher education and income levels. However, among German-speaking women exposed to a regional program, the effect on education was reversed with higher predicted probabilities among women with primary education levels compared to women with tertiary education levels. This effect was not found in the RII/SII analysis which should call for a careful interpretation of the reversal of education inequalities in the context of organized programs.

### Education

Among German-speaking women, mammography programs increased only slightly screening rates, by 3% for women with only a primary school education, and 4% for women with the highest level of education. This suggests that the program did not achieve its goal of motivating women to undergo a mammography among German-speaking women with primary and tertiary education levels. Among Latin women with primary education living in a canton with a mammography program up-to-date screening decreased 12% compared to the opportunistic screening context. In contrast, Latin women with higher education levels benefited significantly more from the implementation of mammography programs (24%) compared to those in cantons with opportunistic screening (10%). In the main analysis education is analyzed as categorical variable (nonlinear) and we find an effect which we do not find with RII/SII. In this sense, the categorical variable analysis better captures the nonlinear relation between education level and screening participation.

### Socioeconomic inequalities

We did not identify socioeconomic inequalities in the Latin region with opportunistic screening. This could be explained by a spillover effect caused by the majority of the Latin region being covered by screening programs (Bulliard *et al.*, 2021). However, among women exposed to an organized program in the Latin region, there were clear income-related inequalities in up-to-date screening. Organized screening programs failing to reduce socioeconomic inequalities is part of a bigger paradox in preventive care, as already observed in Belgium for mammography (Willems and Bracke, 2018). Prevention programs usually perform better in involving patients considered at lower risk and with higher education than those at high risk and from low educational backgrounds, thereby potentially widening the inequality gap in health disparities (Capewell and Graham, 2010).

Overall, we observed that income level continues to be an obstacle for up-to-date breast cancer screening in both German and Latin regions and that programs do not reduce income-related inequalities in mammography screening. This could be due to the cost of screening and the overall high out-of-pocket health expenditures in Switzerland (Penders *et al.*, 2017). Although personal health insurance covers 90% of the cost in opportunistic regions, there is a remaining 10% of approximately 20 CHF. Moreover, each health insurance policy has a yearly deductible ranging from 300 CHF to 2500 CHF, which patients fully pay before the insurance covers any expense. Screening also includes indirect costs like missing time at work or finding childcare for mothers. All those extra direct and indirect costs likely prevent women living in low-income households from undergoing mammography (Guessous *et al.*, 2012; Harvey *et al.*, 2014). Although screening programs can encourage women to undergo a mammography (Pletscher, 2016), our study aligns with previous findings indicating that screening only partly reduces socioeconomic inequalities in breast cancer prevention (Sandoval *et al.*, 2017; Cullati *et al.*, 2018).

### Screening programs models

For any prevention campaign to be successful, there is a need for a clear message, that reaches the targeted population, and is expressed in a way that is understandable to the target population. Based on this model, there could be multiple reasons for a lack of benefit of mammography screening programs on socioeconomic inequalities in attendance. The screening campaigns may have failed to address the worries of women regarding mammography. Applying specific communication strategies is challenging, as regional programs rely on a ‘one-size-fits-all’ approach (Giordano *et al.*, 2005; Relecom *et al.*, 2018). Moreover, women with primary education level are more likely to have a lower health literacy (Stormacq *et al.*, 2019), and are more likely to have a migration background than women with secondary or tertiary education (Pasick and Burke, 2008) which might influence their attitude toward preventive health measures.

Switzerland was a special case due to the controversy surrounding mammography programs. In 2013, the Swiss Medical Board recommended discontinuing mammography programs after having conducted a synthesis of the scientific evidence on the benefits and harms of mammography (Swiss Medical Board, 2013), sparking a public debate reported by the Swiss media. Public health researchers disagreed with this recommendation and argued for the continuation of mammography programs (Biller-Andorno and Jüni, 2014; Chiolero and Rodondi, 2014). As a result, no mammography programs were stopped, and Switzerland continued to implement new programs in different regions of the country. This controversy may, thus, have minimally influenced attitudes of the general population toward mammography screening.

In the German region, however, screening programs partially reversed the association between education and screening rates and increased the proportion of screening among primary educated women. This effect might be caused by a different cultural understanding of prevention in general, and cancer prevention between the German and Latin regions of Switzerland. Another explanation might be that since the screening campaigns are organized at the canton level and not at the national level the German region might have elaborated better strategies to reach socio-economically disadvantaged women or might have a better engagement of local actors into the programs. Further studies into the nature of each local screening campaign will be necessary to establish whether this observed difference results from a cultural difference or a difference in the efficacy and implementation of mammography screening campaigns.

### Strengths and limitations

Our sample may not be fully representative of women aged 50–69 in Switzerland, as the original sampling of the SHIS was designed to represent people aged 15 and older living in Switzerland. Furthermore, the declining response rates going from 84.5% to 51%, despite being on par with other studies, are likely to have accentuated any potential selection bias. The measurement bias is inherent to the self-reported nature of the outcome variable. Measurement bias might also come from our modifier variables which may contain cantonal variations in the implementation of programs such as their practical organization, recruitment, and financial participation of the patients. For example, some cantons requested 10% of financial participation to the total cost from patients, some offered provisory free participation, and some are completely free of charge. Similarly, our second modifier variable, the language spoken in the municipality of residence, only served as a proxy for cultural differences. Thus, it may not capture the full extent of granular differences between diverse cultures in Switzerland particularly regarding people with a migration background, as well as the impact of religion or education. Finally, there are additional limitations to be considered given that our data did not follow the same individuals over time, exposure was not completely at random, and the assumption of stable unit treatment value was probably threatened, because women not exposed to mammography programs were probably aware of the existence of these programs in other Swiss regions.

The study’s main strength is the large survey administration. Each cross-sectional survey is designed to ensure national representativeness through a two-stage large random sampling process, drawing participants from the general population register and a standardized data collection through computer-assisted telephone interviews, complemented by written questionnaires that are available in German, French, and Italian.

### Conclusion

Socioeconomic inequalities persist in mammography uptake, especially in opportunistic screening. Regional screening programs only reduce these inequalities in German regions, namely by negating the effect of education on up-to-date mammography rates. Public health agencies should consider potential cultural understandings of preventive exams inherent to Latin and Germanic cultures when formulating strategies regarding breast cancer screening programs. When designing prevention initiatives in multicultural countries, addressing the different cultural perceptions and attitudes toward programs could ensure that prevention initiatives are not only effective but also take the cultural factor across different socioeconomic groups into account. This might imply moving away from one-dimensional screening programs to adopting a multimodal and culturally tailored approach, which would better fit with prevention models for vulnerable population.

## Acknowledgements

This work was supported by a grant from Swiss Cancer Research (HRS-4082-11-2016).

M.v.A., C.P.B., I.G., D.S.C., and S.C. conceived and designed the study. V.J. analyzed the data. C.P.B. and M.v.A. wrote the paper. All authors reviewed and were involved in writing the manuscript.

This study was part of the Swiss Health Interview Survey, which is under the responsibility of the Swiss Federal Statistical Office and was approved by the relevant research ethics committees in Switzerland. Data protection and individual privacy are regulated by the Federal Statistics Act of 1992.

This study used the data from the Swiss Health Interview Survey, a public database. The data are available for a fee and users must obtain permission from the Swiss Federal Statistical Office (gesundheit@bfs.admin.ch).

During the preparation of this work the authors used DeepL Write in order to check the English grammar and readability. After using this tool/service, the authors reviewed and edited the content as necessary and take full responsibility for the content of the publication.

### Conflicts of interest

There are no conflicts of interest.
